# MART-1 peptide vaccination plus IMP321 (LAG-3Ig fusion protein) in patients receiving autologous PBMCs after lymphodepletion: results of a Phase I trial

**DOI:** 10.1186/1479-5876-12-97

**Published:** 2014-04-12

**Authors:** Emanuela Romano, Olivier Michielin, Verena Voelter, Julien Laurent, Hélène Bichat, Athina Stravodimou, Pedro Romero, Daniel E Speiser, Frédéric Triebel, Serge Leyvraz, Alexandre Harari

**Affiliations:** 1 Department of Oncology, Service of Medical Oncology, CHUV BH-06 1011 Lausanne, Switzerland; 2 Laboratory of tumor immunobiology, Department of Oncology, University Hospital of Lausanne, Lausanne, Switzerland; 3 Ludwig Center for Cancer Research at the University of Lausanne, Lausanne, Switzerland; 4 Center of Experimental Therapeutics, Department of Oncology, University Hospital of Lausanne, Lausanne, Switzerland; 5 Immutep, SA, Orsay, France; 6 Current address: Merck-Serono, Darmstadt, Germany

**Keywords:** Melanoma, Immunotherapy, Immunization, Adjuvant, Immunogenicity, LAG-3Ig, Tumor-specific CD8 T cells, Adoptive cell therapy

## Abstract

**Background:**

Immunotherapy offers a promising novel approach for the treatment of cancer and both adoptive T-cell transfer and immune modulation lead to regression of advanced melanoma. However, the potential synergy between these two strategies remains unclear.

**Methods:**

We investigated in 12 patients with advanced stage IV melanoma the effect of multiple MART-1 analog peptide vaccinations with (n = 6) or without (n = 6) IMP321 (LAG-3Ig fusion protein) as an adjuvant in combination with lymphodepleting chemotherapy and adoptive transfer of autologous PBMCs at day (D) 0 (Trial registration No: NCT00324623). All patients were selected on the basis of *ex vivo* detectable MART-1-specific CD8 T-cell responses and immunized at D0, 8, 15, 22, 28, 52, and 74 post-reinfusion.

**Results:**

After immunization, a significant expansion of MART-1-specific CD8 T cells was measured in 83% (n = 5/6) and 17% (n = 1/6) of patients from the IMP321 and control groups, respectively (*P* < 0.02). Compared to the control group, the mean fold increase of MART-1-specific CD8 T cells in the IMP321 group was respectively >2-, >4- and >6-fold higher at D15, D30 and D60 (*P* < 0.02). Long-lasting MART-1-specific CD8 T-cell responses were significantly associated with IMP321 (*P* < 0.02). At the peak of the response, MART-1-specific CD8 T cells contained higher proportions of effector (CCR7^−^ CD45RA^+/−^) cells in the IMP321 group (*P* < 0.02) and showed no sign of exhaustion (*i.e.* were mostly PD1^−^CD160^−^TIM3^−^LAG3^−^2B4^+/−^). Moreover, IMP321 was associated with a significantly reduced expansion of regulatory T cells (*P* < 0.04); consistently, we observed a negative correlation between the relative expansion of MART-1-specific CD8 T cells and of regulatory T cells_._ Finally, although there were no confirmed responses as per RECIST criteria, a transient, 30-day partial response was observed in a patient from the IMP321 group.

**Conclusions:**

Vaccination with IMP321 as an adjuvant in combination with lymphodepleting chemotherapy and adoptive transfer of autologous PBMCs induced more robust and durable cellular antitumor immune responses, supporting further development of IMP321 as an adjuvant for future immunotherapeutic strategies.

## Background

Recent advances in the understanding of the complex cellular interactions regulating cancer immunity have led to new strategies in the development of cancer immunotherapy. There is strong evidence that both adoptive T-cell transfer and T-cell checkpoint blockade can lead to regression of advanced melanoma [1-4]. Adoptive cell therapy using tumor-infiltrating lymphocytes (TILs) can mediate objective and durable tumor regressions in patients with metastatic melanoma; however, melanoma samples cannot be obtained for TIL production from all patients and, in some cases, TIL cannot be isolated from the resected tumor. To overcome those potential limitations, we capitalized on the adoptive transfer of peripheral blood mononuclear cells (PBMCs), with the hypothesis that the vaccine would prime newly infused T cells to mediate tumor response.

In murine models and in early clinical trials, lymphodepletion seemed to enhance the antitumor effects of transferred T cells *in vivo* by several mechanisms including the elimination of suppressive regulatory T lymphocytes (Tregs) [5], the elimination of cellular “sinks” for homeostatic cytokines such as IL-7 and IL-15 [6], and the engagement of toll-like receptors on antigen-presenting cells after damage of the gut epithelium [7,8]. In these murine models, there was a direct relationship between the extent of lymphodepletion and the magnitude of the *in vivo* antitumor effect of the transferred cells.

In our prior clinical trial [9,10], patients with advanced stage III/IV melanoma received a lymphodepleting, nonmyeloablative chemotherapy consisting of Busulfan and Fludarabine before adoptive transfer of autologous PBMCs and MART-1 analog peptide vaccination. This conditioning regimen induced a suboptimal lymphodepletion at the time of cell infusion and was associated to a prolonged lymphopenia affecting long-term immune reconstitution. We reported a long-lasting (over 2 years) objective response in 1 out of 6 patients [9]. In a subsequent clinical phase I trial, we tested whether the use of a different lymphodepleting regimen of Cyclophosphamide (CTX) at 30 mg/kg or at 60 mg/kg plus Fludarabine at 30 mg/m^2^ followed by adoptive transfer of autologous PBMCs and vaccination with MART-1peptide emulsified in Incomplete Freund’s Adjuvant (IFA) would increase the expansion *in vivo* of tumor-specific T cells and induce a stronger anti-tumor protection [10]. We showed that CTX plus Fludarabine was superior to Busulfan plus Fludarabine conditioning in terms of degree of lymphodepletion, with a maximal effect obtained with a CTX dose of 60 mg/kg, and that reconstitution of T cells, particularly of CD8 T cells, was more rapid. We reported that the depth of homeostatic T-cell proliferation correlated tightly with the extent of lymphodepletion and was accompanied by increased levels of IL-15 but not of IL-7; however, despite efficient homeostatic proliferation of total CD4 and CD8 T cells, the frequency of CD8 T cells specific for MART-1 and cancer-testis antigens were quite low. In contrast, we observed a substantial proliferation of EBV-specific T cells; whether this was due to homeostatic proliferation or viral reactivation remains to be established [10].

Another question that remains so far unanswered is whether the association of tumor-peptide vaccination combined with a stronger adjuvant after adoptive cell transfer would induce a more sustained anti-tumor specific CD8 T-cell expansion and potentially counterbalance the homeostatic proliferation of Tregs *in vivo*. A large body of work supports the idea that immunogenicity of a vaccine preparation is critical to induce measurable antigen-specific T-cell responses *in vivo*. Vaccines containing peptides mixed with IFA alone elicit only moderate CD8 T lymphocyte-mediated responses [11].

Lymphocyte activation gene-3 (LAG-3 or CD223) protein is an important regulator of T-cell homeostasis [12]. It is a non-TLR (toll-like receptor) activator of antigen-presenting cells (APCs) and induces dendritic cells (DCs) migration and antigen cross-presentation to CD8 T cells. It binds to MHC class II molecules that normally present antigenic peptides to CD4 T cells. LAG-3 is evolutionarily related to CD4 and has retained an affinity of 2 logs higher than CD4 for their common ligand, MHC II molecules on APCs such as dendritic cells. A soluble form of the LAG-3 (sLAG-3) molecule that is derived from an alternative splicing event can be found in human serum. In a study, breast cancer patients who had high levels of sLAG-3 in their sera at diagnosis were shown to have a better overall survival than patients with low sLAG-3 levels [13].

Based on these observations, a dimeric recombinant IMP321 molecule has been produced cGMP, consisting of the four LAG-3 extracellular Ig-like domains fused to the Fc fraction of a human IgG1 (LAG-3Ig). IMP321 acts as an APC activator and is a novel adjuvant that induces potent antigen-specific CD8 T-cell responses. Non-TLR agonists like CD40L or LAG-3Ig are human proteins that promote DC maturation and migration [14-17], as well as to support the antigen-cross-presentation to a greater extent than other commonly used adjuvants [18]. In the present study, we set out to address the question whether the combination of lymphodepleting chemotherapy of CTX at 30 mg/kg followed by Fludarabine at 30 mg/m^2^ and adoptive transfer of autologous PBMCs in association to a MART-1 peptide vaccination with or without the presence of the adjuvant IMP321 would elicit a more robust and long-lasting anti-tumor immunity that would finally improve patient outcome. Our study showed a more robust and durable expansion of MART-1-specific CD8 T cells in the IMP321 group, associated to only transient clinical response in one patient.

## Methods

### Study groups

The present trial was approved by the local IRBs and by the Swiss national regulatory authority (Swissmedic). It has been registered in the NCI Clinical Trials PDQ under the number NCT00324623. Patients with stage IV cutaneous melanoma were accrued between October 2005 and August 2011. Eligibility was based on the following inclusion criteria: progressive disease with no evidence of brain metastases, HLA-A2 positivity; tumor expression of Melan-A by IHC; and a measurable, endogenous anti-MART-1 CD8 T-cell response for patients that had no prior adjuvant vaccine therapy, as described [10]. MART-1 CD8 T-cell responses were assessed by tetramer staining using ELAGIGILTV-MHC class I multimers with a threshold of 0.03% of CD8 T cells above background. Patients underwent CT scan of the thorax, abdomen, and pelvis within 4 weeks of therapy. After collection of peripheral blood mononuclear cells (PBMCs) by lymphocytapheresis, patients received alymphodepleting chemotherapy. Patients received 2 days (D-7 and D-6) of CTX at 30 mg/kg, followed by 3 days (D-5 to D-3) of Fludarabine at 30 mg/m^2^. Three days later (D0), cryopreserved PBMCs (8.9 ± 0.86×10^9^) with a mean 90% viability were reinfused (individual patient’s numbers are provided in Additional file 1: Table S1), and peptide vaccination with the MART-1 analog peptide (MART-1_26–35_, A27L (ELAGIGILTV), LICR Melbourne-Austin Branch) emulsified in Incomplete Freund's Adjuvant IFA-51 (IFA; Seppic Inc, France) was given subcutaneously (s.c.) at D8, 15, 22, 28, 52, and 74 post-reinfusion as described [11,19]. IMP321 (LAG-3Ig fusion protein, Immutep S.A., Orsay, France) was added to the vaccine for 6 of the 12 patients, at a final concentration of 25 μg (3 patients) and 250 μg (3 patients). Blood samples for immunomonitoring were collected before each vaccine. G-CSF at 5 μg/kg was given s.c. starting at D1 until absolute neutrophil counts (ANC) reached > 1000 cells/μL. All patients received at least 6-month of TMP/SMX prophylaxis and until CD4 T-cell counts were > 200 cells/μL. Patients with positive serology for HSV at baseline received antiviral prophylaxis with valaciclovir until recovery of neutropenia (ANC > 1000 cells/μL).

### Synthetic peptide and peptide-MHC class I multimer complex

HPLC-purified (>80% purity) MART-1 analog peptide (MART-1_26–35_, A27L, ELAGIGILTV) was obtained from LICR (Melbourn-Austin Branch) and ELAGIGILTV-MHC class I multimers were purchased from ProImmune (Oxford, UK). HLA-A2 restricted Influenza (Flu, GILGFVFTL), Epstein-Barr Virus (EBV, GLCTLVAML) and Cytomegalovirus (CMV, NLVPMVATV) peptides were purchased from JPT (JPT Peptide Technologies GmbH, Germany).

### Antibodies

The following Abs were used in different combinations. CD8-PB, CD8-APCH7, CD3-APCH7, CD45RA-PECy5, PD-1-FITC, IFN-γ-APC, TNF-α-PECy7, and IL-2-PE were purchased from Becton Dickinson (BD, San Diego, CA), CD4-ECD, CD3-ECD, CD28-ECD, CD27-APC from Beckman Coulter (Fullerton, CA, USA), perforin-FITC (clone B-D48) from Diaclone (Besançon, France), CCR7-FITC from R&D Systems (Minneapolis, MN, USA), 2B4-PE-Cy5.5 and CD160-APC from Biolegend (San Diego, CA, USA) and CD127-PE-Cy7 from eBioscience (San Diego, CA, USA).

### Flow cytometer

All flow cytometry analyses from the study were performed on an LSRII Becton Dickinson (BD, San Diego, CA) SORP (4 lasers). Daily maintenance of the flow cytometer and optimal settings were determined using BD CS&T bright bead target values for each fluorescence detector, to obtain consistent and reproducible results over time.

### *Ex vivo* analysis of tumor-specific CD8 T cells

Cryo-preserved blood mononuclear cells (1-2×10^6^) cells were stained for dead cells (Aqua LIVE/DEAD, Invitrogen) and then stained with appropriately tittered peptide-MHC class I multimer complexes at 4°C for 30’ in Ca^2+^-free media as described [20]. Cells were then washed and directly stained at 4°C for 20’ with the following Abs in various combinations: CD3, CD8, CD45RA, CD127, CCR7, CD28, CD27, PD-1, 2B4, CD160. Finally, cells were fixed (CellFix, BD), acquired on an LSRII SORP and analyzed using FlowJo 8.8.2 (Tree star Inc, USA). Analysis and presentation of distributions was performed using SPICE version 5.1, downloaded from <http://exon.niaid.nih.gov/spice>[21]. The number of lymphocyte-gated events ranged between 0.6-1×10^6^ in the flow cytometry experiments.

### ICS assay

Cryo-preserved blood mononuclear cells (1-2×10^6^) were stimulated overnight in 1ml of complete media (RPMI (Invitrogen), 10% fetal bovine serum (FBS; Invitrogen), 100 μg/ml penicillin, 100 unit/ml streptomycin (BioConcept)) in the presence of Golgiplug (1 μl/ml, BD), anti-CD28 (0.5 μg/ml, BD) and 1 μg/ml of peptide as described [22]. Staphylococcus enterotoxin B (SEB; Sigma) stimulation (100 ng/ml) served as positive control. At the end of the stimulation period, cells were stained for dead cells (Aqua LIVE/DEAD, Invitrogen), permeabilized (Cytofix/Cytoperm, BD) and then stained at RT for 20’ with CD4, CD8, CD3, IFN-γ, IL-2, TNF-α and perforin (clone B-D48). Cells were then fixed (CellFix, BD), acquired on an LSRII SORP and analyzed using FlowJo 8.8.2. Analysis and presentation of distributions was performed using SPICE version 5.1, downloaded from <http://exon.niaid.nih.gov/spice>[21].

### IFN-γ ELISpot assay

After 7-day antigen-specific stimulation with HLA-A2+, MART-1_26–35_-loaded T2 cells at 1:10 APC:T cell ratio, responder lymphocytes were tested for IFN-γ production by ELISpot assay (1-DIK ELISpot for human IFN-γ, Mabtech, DiaPharma Group; Vector ABC kit, Vector Laboratories; Automated ELISpot Bioreader 5000, BIO-SYS GmbH) according to the manufacturer’s instructions. Targets for lymphocyte restimulation during overnight rechallenge in the ELISpot assays were either MART-1_26–35_-pulsed, unpulsed T2, or HLA-A2+, MART-1+ melanoma Me205 cell lines, plated in triplicates at 30:1 effector:target (E:T) ratio. Control wells contained effectors with unloaded T2 targets. Me275 is a human cell line derived from a nodal metastasis of melanoma.

### Functional avidity

Peptide stimulations were performed as described above. Functional avidity of responses was assessed by performing limiting peptide dilutions and determining the peptide concentration required to induce half-maximal IFN-γ responses in *in vitro* assays as described [23]. Peptides were added in serial dilutions ranging from 2 μg/ml to 1 pg/ml. EC_50_ was determined as the peptide concentration needed to achieve a half-maximal response.

### Statistical analysis

Unpaired and paired two-tailed student *t*-tests were performed using Excel (Microsoft, Redmond, WA), or GraphPad Prism version 5.03 (San Diego, CA). Correlations among variables were performed by Spearman’s test. Regarding SPICE analyses of the flow-cytometry data, comparison of distributions was performed using a Student's *t*-test and a partial permutation test as described [21].

## Results

### Demographics and treatment-related toxicity

In this study, we investigated the safety of the combination of lymphodepletion and adoptive cell transfer (ACT) of autologous PBMCs in association to a MART-1-peptide vaccination with or without the presence of the adjuvant IMP321 and whether it would elicit a more robust and long-lasting anti-tumor immunity that could ultimately improve patients outcome. Twelve HLA-A2+ patients with stage IV cutaneous melanoma and *ex vivo* detectable MART-1-specific CD8 T-cell responses were treated. Initial frequencies of circulating MART-1-specific CD8 T cells prior to intervention were balanced between the IMP321 and No IMP321 patient groups (Additional file 2: Figure S1A). Patients were immunized s.c. with a MART-1 peptide at day (D) 0, 8, 15, 22, 28, 52, and 74 post-reinfusion with (n = 6) or without (n = 6) IMP321 as an adjuvant in combination with lymphodepleting chemotherapy and adoptive transfer of autologous PBMCs. Patient gender was balanced in the IMP321 group, as opposed to the no IMP321 group where 66% patients were male (*P* = 0.6, ns). Mean age was 54 (range, 35 to 73 years, *P* = 0.3, ns). ECOG performance status was 0 to 1 for all patients. The majority of patients had stage IV M1a (3 of 6 patients, 50%) and M1c disease (4 of 6 patients, 66%) in the IMP321 and no IMP321 group, respectively. Four patients (66.6%) had prior chemo- or radiotherapy in the IMP321 group and 1 patient (16.6%) in control group, respectively. All patients in this study had previous adjuvant immunotherapy (MART-1-containing anti-tumor vaccines) for their high-risk disease. Demographic data are listed in Table 1. As expected, hematologic toxicity was frequent in both groups with all patients experiencing grade 3–4 leucopenia and CD4 T-cell lymphopenia. Febrile neutropenia was reported in 50% of the patients in the IMP321 group and 33% of the patients in the control group, and resolved without clinical complications. Non-hematological toxicities, as reported in Table 2, were mild.

**Table 1:** Patient Demographics

	**IMP321**	**No IMP321**
	**N (%)**	**N (%)**
**Age**		
Median	49.5	56.8
Range	35-73	48-70
**Gender**		
Male	3 (50%)	4 (66.6%)
Female	3 (50%)	2 (33.3%)
**ECOG PS**		
0	4 (66.6%)	5 (83.3%)
1	2 (33.3%)	1 (16.6%)
**M sub-stage**		
M1a	3 (50%)	1 (16.6%)
M1b	1 (16.6%)	1 (16.6%)
M1c	2 (33.3%)	4 (66.6%)
**Previous therapy for Stage IV**		
Surgery	1 (16.6%)	2 (33.3%)
CHT or RT	2 (33.3%)	1 (16.6%)
Other	2 (33.3%)	0 (0%)
**Adjuvant immunotherapy**		
Yes	6 (100%)	6 (100%)
No	0 (0%)	0 (0%)

**Table 2:** Treatment-related adverse events

	**No IMP321 N (%)**	**IMP321 N (%)**	**No IMP321 N (%)**	**IMP321 N (%)**
**Treatment-related event**				
*Non*-*hematological*	Grade 1-2		Grade 3-4	
Fatigue	6 (100%)	6 (100%)	-	-
Nausea/Vomiting	5 (83%)	1 (16.6%)	-	-
Constipation	-	-	1 (16.6%)	-
Alopecia	6 (100%)	6 (100%)	-	-
Mucositis (oral)	-	1 (16.6%)	-	-
Weight loss	6 (100%)	6 (100%)	-	-
*Hematological*				
Anemia	4 (66.6%)	5 (83%)	1 (16.6%)	1 (16.6%)
Leucopenia	-	-	6 (100%)	6 (100%)
Trombocitopenia	-	4 (66.6%)	4 (66.6%)	2 (33%)
Febrile Neutropenia (grade 3)	n/a	n/a	2 (33%)	3 (50%)
CD4 Lymphopenia (≥ grade 3)	n/a	n/a	6 (100%)	6 (100%)

### Stronger expansion of MART-1-specific CD8 T-cell responses following immunization in the IMP321 group

**Figure 1:**
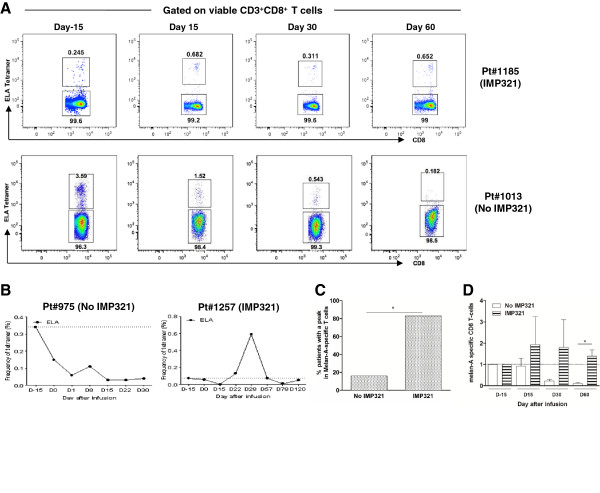
**Kinetic of MART-1-specific CD8 T-cell responses following immunization. A**. Representative example of the increase in the frequency of circulating MART-1-specific CD8 T cells in Pt#1185 and Pt#1013 following infusion. MART-1-specific CD8 T cells are identified using peptide-MHC class I multimer complexes and plots are gated on viable CD3^+^CD8^+^ T cells. **B**. Prototypic examples of the kinetic of MART-1-specific CD8 T cells in patients from the IMP321 or no IMP321 groups. Dotted lines represent the initial frequency of MART-1-specific CD8 T cells prior to lymphodepletion. **C**. Proportion of patients from each group with a peak in the frequency of MART-1-specific CD8 T cells. Peaks were defined as a frequency of MART-1-specific CD8 T cells **>** three-fold higher than baseline, *i.e*. D-15 (n = 6 for each group, *P < 0.02). **D**. Cumulative analysis of the fold increase of MART-1-specific CD8 T cells overtime in the different groups. Mean + SEM are shown (n = 6 for each group, *P < 0.02).

Using peptide-MHC class I multimer complexes, we monitored the frequency of circulating MART-1-specific CD8 T cells on viable CD3^+^ T cells in peripheral blood from patients from both groups (Figure 1A). Analyses were performed at D15, D30 and D60 post-ACT and immunization and were compared to D-15, *i.e*. prior to treatment. As shown in the representative examples from Figure 1A and B, the frequency of MART-1-specific CD8 T cells significantly increased after immunization in the IMP321 group but not in the control (no IMP321) group. Of note, no dose-effect was observed at the 25 μg and 250 μg IMP321 dose levels; therefore, all patients receiving IMP321 are pooled in the present analyses. Cumulative analyses confirmed a significant expansion (defined as three-fold higher than baseline) of MART-1-specific CD8 T cells in 5 out of 6 patients (83%) from the IMP321 group compared to only 1 out of 6 patients (17%) from the control group (*P* < 0.02, Figure 1C). Furthermore, compared to the control group, the mean fold increase of MART-1-specific CD8 T cells was respectively >2-, >4- and >6-fold higher in the IMP321 group at D15, D30 and D60. Consistently with our prior study [10], we observed in the control arm (no IMP321) a progressive and profound decrease in the frequency of MART-1-specific CD8 T cells starting at D15 and still notable at D30 and D60 (Figure 1D). Interestingly, IMP321 appears not only to prevent this drop but also to lead to an increase in the frequency of MART-1-specific CD8 T cells. IMP321 immunization was therefore also associated to the induction of a durable MART-1-specific CD8 T-cell response at D60, which remained higher than baseline and was significantly higher than in the control group (*P* < 0.02, Figure 1D). Of note, absolute leukocyte counts and percentages of CD4 and CD8 T-cells were comparable between the two arms (Additional file 2: Figure S1), thus indicating that the aforementioned differences in the frequency of circulating MART-1-specific CD8 T cells also reflected absolute counts of these cells. Taken together, these observations indicate that vaccination with IMP321 was more immunogenic and lead to a selectively stronger and more durable anti-tumor cellular immune response.

### Immunization with IMP321 induces more effector MART-1-specific CD8 T-cell responses

**Figure 2:**
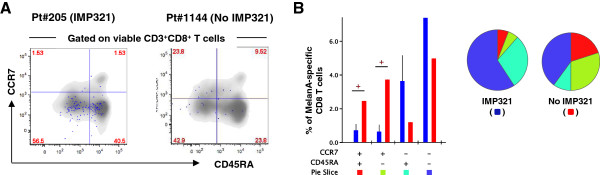
**Analysis of T-cell differentiation of MART-1-specific CD8 T-cell responses following immunization. A**. Representative example of the expression of CD45RA and CCR7 on CD8 T cells in Pt#205 and Pt#1144. Total CD8 T cells appear in grey whereas blue dots represent MART-1-specific CD8 T cells. **B**. Cumulative analysis of the expression of CD45RA and CCR7 on MART-1-specific CD8 T cells in patients from the IMP321 or no IMP321 groups. All possible combinations of the distinct markers are shown on the x axis, whereas the percentages of the distinct cell subsets within MART-1-specific CD8 T cells are shown on the y axis. The pie charts summarize the data, and each slice corresponds to a certain combination of molecules. Stars (+) indicate a significant difference using a two-tailed unpaired student *t* test.

We then performed qualitative analyses of MART-1-specific CD8 T cells at the peak of response for each of the patients from the IMP321 or control groups. We first investigated the level of T-cell differentiation of vaccine-induced MART-1-specific CD8 T cells and analyzed the expression of CD45RA and CCR7. MART-1-specific CD8 T cells from patients of the IMP321 groups were mostly composed of CD45RA^−^CCR7^−^ and of CD45RA^+^CCR7^−^, *i.e*. two subsets of effector cells, whereas significantly higher proportions of CD45RA^+^CCR7^+^ and CD45RA^−^CCR7^+^, *i.e*. naïve and central memory cells, were observed in the control group (Figure 2A-B). These observations indicate that vaccination with IMP321 was selectively associated with the induction of a more effector anti-tumor immunity.

### Functional potency of MART-1-specific CD8 T-cell responses following immunization

**Figure 3:**
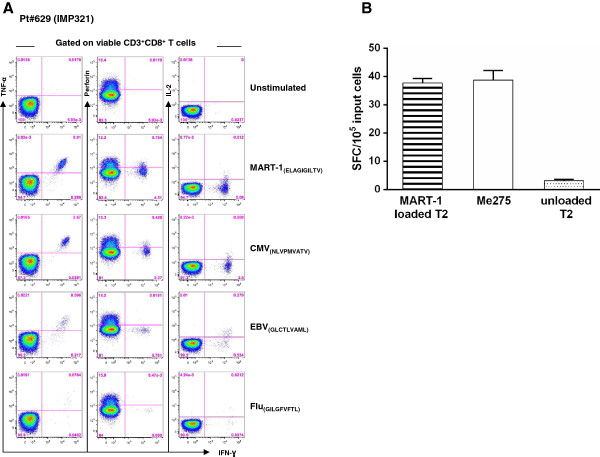
**CD8 T cells at peak of response secrete effector cytokines against viral and tumor Ags. A**. Representative example of the ability of CD8 T cells at peak of response to produce IFN-γ, IL-2 and/or TNF-α and to express perforin by ICS and polychromatic flow cytometry. Profiles are gated on viable CD3^+^CD8^+^ T cells. **B**. PBMCs from melanoma patients at peak of response following ACT and peptide vaccination were restimulated *in vitro* by T2 cells pulsed with MART-1_26–35_ peptide. Following 1 weekly restimulation, ELISpot assays measured IFN-γ secretion after overnight exposure to the respective peptide-loaded (≡), unloaded T2 (░), or Me205 (□) targets. Limited cell numbers precluded isolation of CD3^+^CD8^+^ T cells for the ELISpot assays. Shown are the averaged data (means ± SEM) from 4 independent experiments each one performed in triplicate for the number of IFN-γ spot forming cells (SFC) per triplicate of 10^5^ input cells at 30:1 (E:T) ratio.

We then comprehensively assessed the functional profile and effector function of MART-1-specific CD8 T cells at the peak of responses. For this purpose, cryopreserved PBMC were thawed and stimulated with a panel of cognate tumor- or viral antigens and their ability to produce key cytokines (*i.e.* IFN-γ, IL-2 and/or TNF-α) and to express the cytolytic marker perforin were assessed using polychromatic flow cytometry. Of note, the functional profile of MART-1-specific CD8 T cells was compared to that of an extensive set of virus-specific (*i.e*. Cytomegalovirus [CMV], Epstein Barr virus [EBV] and influenza [Flu]) CD8 T cells. Of interest, as shown in the representative example of Pt#629 from the IMP321 group, MART-1-specific CD8 T cells showed a level of polyfunctionality comparable to that of CMV-specific CD8 T cells (*i.e*. were mostly IFN-γ^+^/TNF-α^+^/IL-2^+/−^/perforin^+/−^; Figure 3A). Conversely, consistently with previous studies [24,25], EBV- and Flu-specific CD8 T cells contained a higher proportion of IL-2-producing cells and showed lower perforin expression as compared to MART-1-specific CD8 T cells (Figure 3A). Furthermore, MART-1-specific CD8 T cells secreted IFN-γ upon recognition of cognate Ag presented at the cell surface of either a peptide-loaded T2 cell line or naturally expressed by the melanoma cell line Me275 (Figure 3B). Taken together, these data demonstrate that MART-1-specific CD8 T cells are polyfunctional and can recognize MART-1-expressing melanoma cells.

### No evidence of T-cell exhaustion of MART-1-specific CD8 T-cell responses following immunization

**Figure 4:**
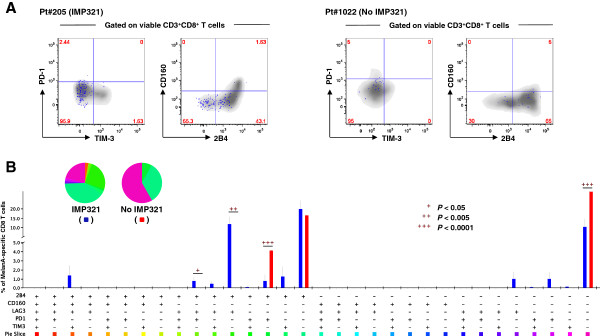
**Analysis of T-cell exhaustion markers of MART-1-specific CD8 T-cell responses following immunization. A**. Representative example of the expression of 2B4, CD160, PD-1, and TIM-3 on CD8 T cells in Pt#205 and Pt#1022. Total CD8 T cells appear in grey whereas blue dots represent MART-1-specific CD8 T cells. **B**. Cumulative analysis of the expression of 2B4, CD160, LAG3, PD-1, and TIM-3 on MART-1-specific CD8 T cells in patients from the IMP321 or no IMP321 groups. All possible combinations of the distinct markers are shown on the x axis, whereas the percentages of the distinct cell subsets within MART-1-specific CD8 T cells are shown on the y axis. The pie charts summarize the data, and each slice corresponds to a certain combination of molecules. Stars (+) indicate a significant difference using a two-tailed unpaired student *t* test.

As a subsequent qualitative analysis, we investigated the level of inhibitory receptor expression (“exhaustion markers”) by vaccine-induced MART-1-specific CD8 T cells. To this end, we analyzed the expression of PD-1, 2B4, CD160, LAG3, and TIM-3 on MART-1-specific CD8 T cells at the peak of response for each of the patients from the IMP321 or control groups. Of interest, the majority of MART-1-specific CD8 T cells in patients from the IMP321 or no IMP321 groups were composed of cells lacking all the markers analyzed (*i.e*. were mostly PD1^−^2B4^−^CD160^−^LAG3^−^TIM3^−^) or only expressed 2B4 (Figure 4A-B). However, as compared to the IMP321 group, a significantly higher proportion of MART-1-specific CD8 T cells from the control group co-expressed PD-1 and 2B4 (Figure 4B). Taken together, these observations indicate that vaccination with IMP321 does not lead to the induction of MART-1-specific CD8 T cells with a typical exhausted phenotype.

### Increase in the functional avidity of MART-1-specific CD8 T cells in patients from the IMP321 group

Functional avidity in an important feature of T-cell immunity [26]. We investigated whether the present experimental strategy would lead to changes in the functional avidity of MART-1-specific CD8 T cells. For this purpose, PBMC were stimulated with limiting dilutions of MART-1 peptides and the functional avidity of CD8 T-cell responses was assessed by determining the peptide concentration required to induce half-maximal IFN-γ responses (*i.e*. EC_50_). Interestingly, when we compared cells isolated at baseline or 2 months after treatment, we observed a significant increase in the functional avidity of MART-1-specific CD8 T cells (*P* < 0.05, Additional file 3: Figure S2).

### Reduced expansion of regulatory T cells following immunization with IMP321

**Figure 5:**
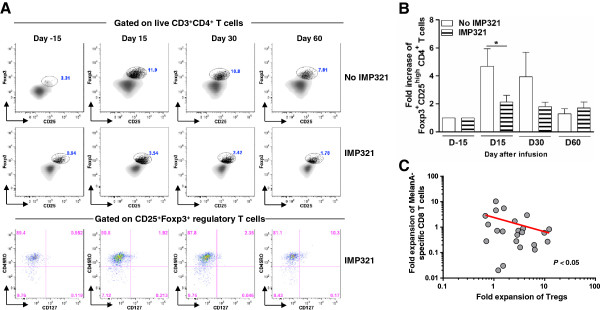
**Analysis of the expansion of regulatory T cells following immunization. A**. Representative examples (top panels) of the frequency of CD25^high^FOXP3^+^ on viable CD3^+^CD4^+^ T cells in patients from the IMP321 or no IMP321 groups. Bottom panels show the expression of CD45RO and CD127 on viable CD3^+^CD4^+^CD25^high^FOXP3^+^ regulatory T cells in a representative patient from the IMP321 group. **B**. Cumulative analysis of the expansion of CD25^high^FOXP3^+^ CD4^+^ T in patients from the IMP321 or no IMP321 groups. The star (+) indicates a significant difference using a two-tailed unpaired student *t* test. **C**. Correlation between the relative expansion of MART-1-specific CD8 T cells and of CD4^+^CD25^high^FOXP3^+^ regulatory T cells (R^2^ = 0.11; *P* < 0.05).

As mentioned above, lymphodepletion may enhance the antitumor effects of transferred T cells by inducing the elimination of suppressive regulatory T lymphocytes (Tregs) [5]. We then analyzed the frequency of Tregs, defined as CD3^+^CD4^+^CD25^high^FOXP3^+^, on live T cells in patients from the IMP321 or control groups. We observed that the frequency of Tregs was significantly higher in the control group compared to the IMP321 group (Figure 5A and B). We then also confirmed that the above gating strategy allowed the identification of true regulatory T cells. As shown in Figure 5A, 80-90% of CD3^+^CD4^+^CD25^high^FOXP3^+^ were CD45RO^+^CD127^−^, thus demonstrating that IMP321 immunization selectively prevented the expansion of adaptive Tregs. Of note, absolute leukocyte counts and percentages of CD4 T-cells were comparable between the two arms (Additional file 2: Figure S1B and C), thus indicating that the aforementioned differences in the frequency of circulating Tregs also reflected absolute counts of these cells.

Finally, we also investigated the potential relationship between the expansion of MART-1-specific CD8 T cells and of Tregs in both groups. Interestingly, we observed a significant negative correlation between the fold increase of MART-1-specific CD8 T cells and that of Tregs (Figure 5C), thus suggesting a potential mechanism for the selective expansion of MART-1-specific CD8 T cells in the IMP321 group.

## Discussion

Current approved therapeutic options in the US and Europe for patients with metastatic melanoma include dacarbazine, interleukin-2, ipilimumab, vemurafenib, dabrafenib, and trametinib, but long-term tumor regression using available agents remains out of reach for most patients. Adoptive cell transfer (ACT) with autologous tumor-infiltrating lymphocytes (TILs) has shown encouraging results in clinical trials, with evidence of durable ongoing complete responses in patients with advanced melanoma. However, melanoma samples cannot be obtained for TIL production from all patients and, in some cases, TILs cannot be isolated from the resected tumor. To overcome those potential limitations, we capitalized on the adoptive transfer of PBMCs in combination with a MART-1 peptide vaccination supplemented or not with IMP321. In this phase I study we included a total of twelve, previously treated, advanced melanoma patients. All the patients were HLA-A*0201 positive and had a detectable frequency of MART-1-reactive T cells at baseline. Age and gender were balanced between groups, as opposed to disease stage distribution with a preponderance of subjects with worst prognosis (66.6% *vs* 33.3% stage IV M1c) in the no IMP321 group. Similar to previously reported ACT studies, the toxicities reported in our trial were mostly hematological and related to the lymphodepleting conditioning regimen.

We set a high threshold (3-fold increase from baseline) to define a MART-1-specific immune response, and IMP321-supplemented vaccines stimulated significantly greater and more sustained MART-1 tetramer reactivity than the control group, suggesting that the addition of a vaccine adjuvant such as the IMP321 is essential for boosting anti-tumor immunity. No systemic dose-effect was observed, however, at the 25 μg and 250 μg IMP321 dose levels, because such low doses were expected to elicit local or loco-regional activation of skin APCs; whereby, an IMP321 dose of 6 mg promoted a systemic effect in a pharmacokinetic study in patients with advanced renal cell carcinoma [27]. Our data also show that IMP321 is essential to prevent the decrease in tumor-specific CD8 T cells observed without IMP321 [10]. These observations are in agreement with previous studies, showing increased CD8 T cells and NK cell production of Th1-effector cytokines such as IFN-γ and/or TNF-α [28] and increased anti-tumor responses in patients with metastatic breast cancer treated with paclitaxel in combination with IMP321 [29]. Our data also showed that IMP321 supported the long-term expansion of tumor antigen-specific CD8 T cells with effector phenotypes including CD45RA^−^CCR7^−^ and of CD45RA^+^CCR7^−^ cells. Antigen-experienced T cells comprise both effector memory (T_EM_) and central memory (T_CM_), which home respectively to inflamed tissues and lymphoid organs [30]. T_EM_ cells are associated with development of a more robust, short-lived anti-tumor immunity [31].

Over the past decade, an overwhelming number of studies highlighted the association between the expression, and in particular the co-expression, of co-inhibitory receptors and a state of dysfunction or anergy also called exhaustion, as found in infectious diseases [26,32], and in cancer [33]. These pathways are also exploited by tumors to induce immune tolerance. In our study, we monitored the expression of a large panel of inhibitory receptors and, despite minor differences between groups, found no evidence of co-expression of those receptors on circulating T cells, thus indicating the lack of exhaustion of MART-1-specific CD8 T-cell responses following immunization, both in the IMP321 and the control arm.

Of note, functional avidity is also a relevant component of CD8 T cells, as it reflects their sensitivity to cognate antigens, *i.e*. how prone T cells are to respond when they encounter low doses of antigen. It is well established that high-avidity CD8 T-cell responses are of higher efficacy against cancers [34]. Several studies aimed at increasing the avidity of anti-tumoral T cells [35,36]. Of interest, in the present study, we observed an increase in the functional avidity of MART-1-specific CD8 T cells after ACT of autologous cells and immunization. Unfortunately, our data do not allow us to establish the influence of the multiple immunizations (as compared to single immunization, for instance) nor the potential contribution of IMP321. The aforementioned increase in CD8 T-cell avidity upon immunization, however, is consistent with a recent study showing increase in T-cell avidity in HIV infection following antigen re-exposure (virus rebound) [23].

Similar to our experience with the MART-1 vaccine, many other cancer vaccines have also failed to induce measurable clinical benefit, often despite the induction of seemingly potent tumor antigen-specific Teff responses [37,38]. There are many potential explanations for this, but one that has received particular attention in recent years emphasizes the role of CD4^+^CD25^+^FoxP3^+^ Tregs. Tregs play a central role in tempering immunity and in maintaining immune homeostasis. However, a growing body of evidence supports the concept that Tregs can also block the generation of effective anti-tumor immunity [39]. It is now well established that CD4^+^CD25^+^Foxp3^+^ Tregs encompass two categories of lymphocytes, natural and adaptive (or induced) Tregs. Until recently, evidence for the recognition of tumor antigens by Treg had been scarce, and it was unclear whether or not Tregs would be activated and expand in response to vaccination against tumor antigens. In recent years, however, a number of reports have identified Tregs specific for a range of tumor antigens in human cancer, including NY-ESO-1, survivin, TRP-1, gp100, MAGE-A3, and MART-1 [40]. Adaptive Tregs have been shown to develop *in vivo* following suboptimal antigen stimulation, in situations characterized by chronic inflammation, in the context of autoimmunity, allergy, and immune responses to tumors. In our study, we observed that the frequency of adaptive Tregs was significantly higher in the control group, compared with the group vaccinated with the same immunogen plus the adjuvant IMP321 and that their frequency inversely correlated with the expansion of MART-1-specific CD8 T cells. We speculate that such an increased Treg frequency in the control group may be due to the suboptimal antigenic stimulation provided in the MART-1 plus IFA51 vaccination in immune compromised hosts, which likely lead to a tolerogenic rather than tumor-specific response against MART-1. On the contrary, provision of the IMP321 to the vaccine was more immunogenic and resulted in significant increase of tetramer reactive MART-1 CD8 T cells with an effector phenotype and a decreased frequency of adaptive Tregs. This observation is in line with recent findings in a melanoma mouse model of peptide vaccination, which found that antigen-specific Tregs were induced following subcutaneous vaccination with either OVA or melanoma-derived peptides, with a restricted expansion of effector T cells. Whereas, the addition of the more potent adjuvants CpG-ODN or Poly (I: C) preferentially amplified effector T cells over Tregs [41].

Although the provision of IMP321 contributed to a significant increase of tetramer reactive MART-1 CD8 T cells with effector properties and a decreased frequency of adaptive Tregs, only one out of 6 patients in the IMP321 cohort achieved a short-lived PR (Additional file 4: Figure S3) that could not be confirmed at 24-weeks post treatment (as per RECIST criteria v1.1). One likely explanation for the low clinical activity observed in this study might rely on the use of non-tumor specific PBMCs instead of TILs, as effector cells in lymphodepleted hosts. Several studies have demonstrated superior anti-tumor reactivity *in vitro* and *in vivo* of TILs as compared with MART-1–specific CTL lines expanded from PBMCs [1,42,43]. In addition, lymphodepleting chemotherapy could have ablated antigen presenting cells, including skin dendritic cells, countering the potential effect of low-dose IMP321 on efficient antigen-presentation and T-cell priming at the vaccine site, thus hindering the clinical efficacy of our vaccination strategy. The use of vaccines based on tumor-loaded DCs [44] or short- plus long-peptide mix to favor helper CD4^+^ T-cell responses could further improve immunogenicity and clinical efficacy. Among the outstanding challenges, foremost is the need to develop ACT strategies that are readily applicable and less labor-intensive. The recent development of faster and optimized protocols for TILs isolation and expansion should allow broader application of TILs-based approaches. Emerging techniques to engineer T-cell receptors (TCRs) or chimeric antigen receptors (CARs) using lymphocytes from peripheral blood may also offer new avenues in ACT and should result in increased clinical benefit.

## Conclusions

The present regimen comprising multiple MART-1 vaccinations with or without IMP321 as an adjuvant in combination with lymphodepleting chemotherapy and adoptive transfer of autologous PBMCs was safe and immunogenic. In the arm receiving IMP321 we observed more robust and durable cellular antitumor immune responses, therefore we encourage further development of IMP321 for future immunotherapeutic strategies.

## Abbreviations

IMP321: LAG-3Ig fusion protein; TILs: Tumor-infiltrating lymphocytes; PBMCs: Peripheral blood mononuclear cells; Tregs: Regulatory T lymphocytes; CTX: Cyclophosphamide; TLR: Toll-like receptor; DC: Dendritic cells; APC: Antigen-presenting cells; EC50: Effect concentration 50%; ACT: Adoptive cell therapy; MART-1: MelanA/MART-1.

## Competing interests

FT is an employee and stock holder of Immutep S.A.

ER, OM, BH, AS, DES, VV, JL, PR, SL, and AH have no financial competing interests to disclose.

## Authors’ contributions

Conception and design: SL, VV, JL, DES, and PR. Writing of the manuscript: ER, SL, and AH. Data analysis: ER, HB, AS, SL, and AH. Patients’ recruitment: VV, OM, ER, and SL. All authors have read and approved the manuscript.

## Supplementary Material

Additional file 1: Table S1 Absolute numbers of PBMCs collected and infused for each patient. Click here for file

Additional file 2: Figure S1 MART-1-specific CD8 T cells and T-cell counts at baseline and post-treatment. Frequencies of MART-1-specific CD8 T cells **(A)**, total leukocyte counts **(B)**, percentages of CD4 **(C)** and CD8 **(D)** T cells are shown prior to treatment (*i.e*. Bsl: baseline) and/or at day (D) 0, 15, 30, and 60 after PBMC infusion. Shown are median + interquartile ranges in panel A and the mean ± SD in panels **B-D**. Patients from the IMP321 and no IMP321 groups are shown in blue circles and red squares, respectively. Click here for file

Additional file 3: Figure S2 Analysis of the functional avidity of MART-1-specific CD8 T cells following immunization plus IMP321. **A**. Representative example of the functional avidity of MART-1-specific CD8 T cells prior to (blue circles) and then 60 days after (red triangles) ACT and immunization. PBMC were stimulated with decreasing concentration of MART-1 peptide and the frequency of IFN-γ-producing CD8 T cells was determined by ICS. The dashed line corresponds to half of the maximal response allowing the extrapolation of the 50% effect concentration (EC_50_). **B**. Cumulative analysis showing the significant increase in the functional avidity of MART-1-specific CD8 T cells following immunization (n = 3). Click here for file

Additional file 4: Figure S3 Short-lived partial response in a patient from the IMP321 group. Panel **A**, baseline thorax-abdomen CT scan. Panel **B**, thorax-abdomen CT scan 30 days after completion of study treatment. Click here for file
