# Supplementary material for: MART-1 peptide vaccination plus IMP321 (LAG-3Ig fusion protein) in patients receiving autologous PBMCs after lymphodepletion: results of a Phase I trial
Source: J Transl Med. 2014 Apr 12;12:97. doi: 10.1186/1479-5876-12-97 (PMC4021605; doi:10.1186/1479-5876-12-97)
Supplement: Additional file 1: Table S1 — Absolute numbers of PBMCs collected and infused for each patient. [file 1479-5876-12-97-S1.doc]

**Suppl. Table 1. Absolute numbers of PBMCs** **collected and infused for each patient.**

| **Patient #** | **PBMCs**  **from apheresis [x10^9^]** | **Infused viable PBMCs [x10^9^]** |
| --- | --- | --- |
| **No IMP321** | | |
| 975 | 5.7 | 5.2 |
| 1013 | 10.9 | 9.7 |
| 1022 | 13.2 | 11.3 |
| 936 | 6.8 | 5.7 |
| 1013 | 7.5 | 6.5 |
| 07 | 11.2 | 10.8 |
| **IMP321** | | |
| 1144 | 8.8 | 8.8 |
| 205 | 17.8 | 15.9 |
| 629 | 12.8 | 10.7 |
| 1131 | 8.4 | 8.3 |
| 1257 | 7.6 | 6.8 |
| 1185 | 8.8 | 7.4 |
