# Supplementary figures and images for: MART-1 peptide vaccination plus IMP321 (LAG-3Ig fusion protein) in patients receiving autologous PBMCs after lymphodepletion: results of a Phase I trial
Source: J Transl Med. 2014 Apr 12;12:97. doi: 10.1186/1479-5876-12-97 (PMC4021605; doi:10.1186/1479-5876-12-97)

Suppl. Fig. 1

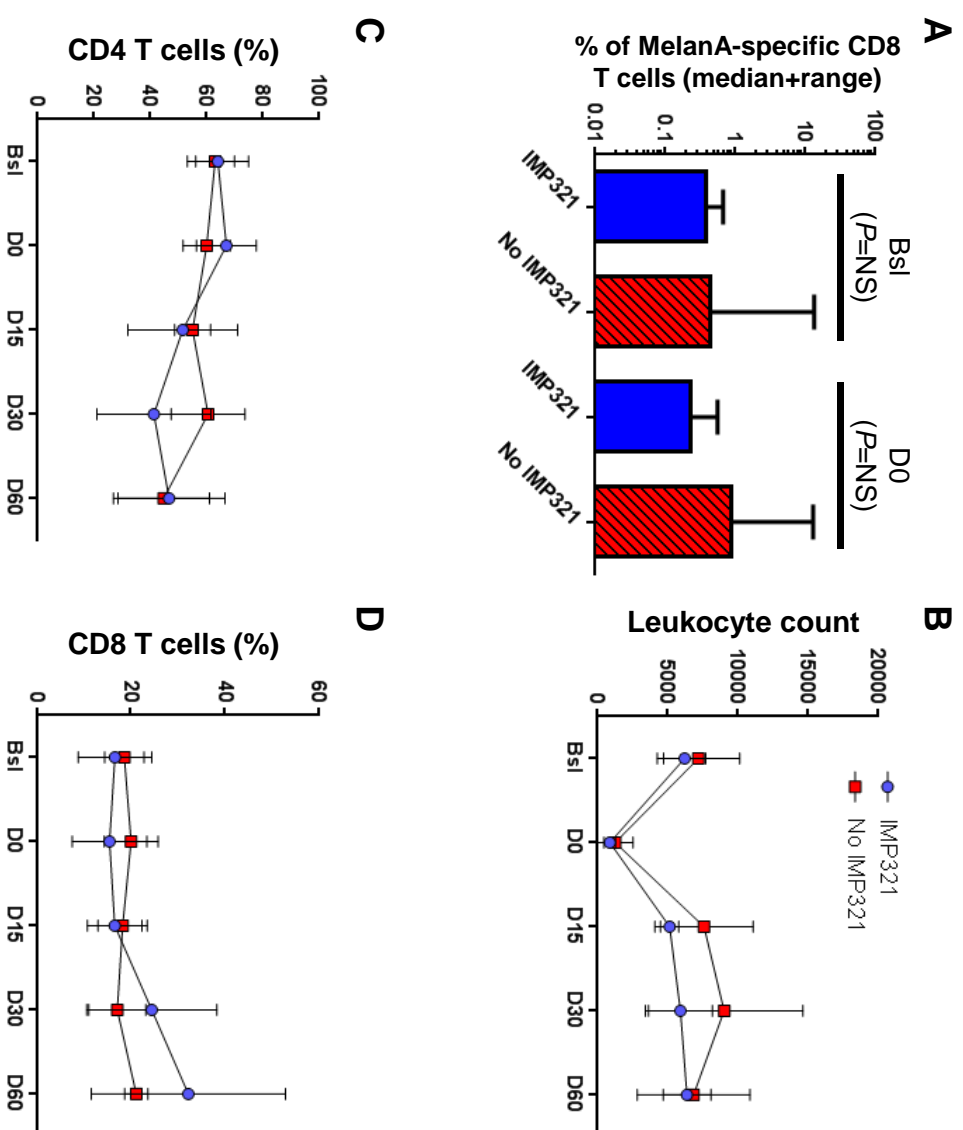

Supplement: Additional file 2: Figure S1 — MART-1-specific CD8 T cells and T-cell counts at baseline and post-treatment. Frequencies of MART-1-specific CD8 T cells (A), total leukocyte counts (B), percentages of CD4 (C) and CD8 (D) T cells are shown prior to treatment (i.e. Bsl: baseline) and/or at day (D) 0, 15, 30, and 60 after PBMC infusion. Shown are median + interquartile ranges in panel A and the mean ± SD in panels B-D. Patients from the IMP321 and no IMP321 groups are shown in blue circles and red squares, respectively. [file 1479-5876-12-97-S2.pdf]

Suppl. Fig. 2

**A**

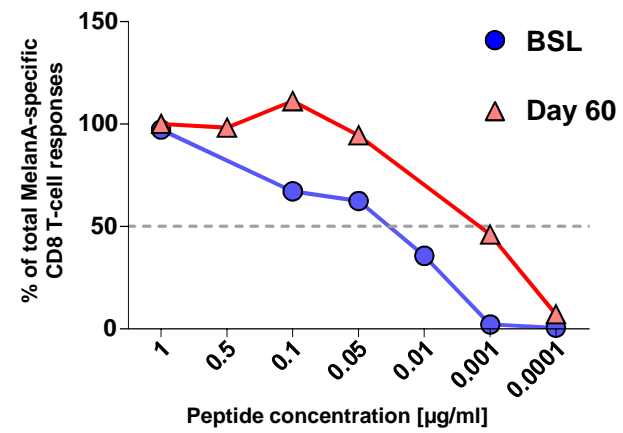

**B**

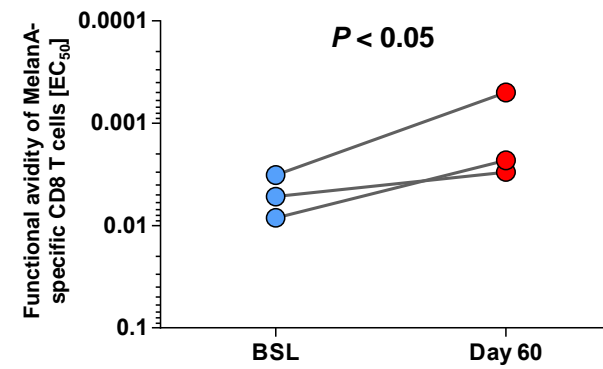

Supplement: Additional file 3: Figure S2 — Analysis of the functional avidity of MART-1-specific CD8 T cells following immunization plus IMP321. A. Representative example of the functional avidity of MART-1-specific CD8 T cells prior to (blue circles) and then 60 days after (red triangles) ACT and immunization. PBMC were stimulated with decreasing concentration of MART-1 peptide and the frequency of IFN-γ-producing CD8 T cells was determined by ICS. The dashed line corresponds to half of the maximal response allowing the extrapolation of the 50% effect concentration (EC50). B. Cumulative analysis showing the significant increase in the functional avidity of MART-1-specific CD8 T cells following immunization (n = 3). [file 1479-5876-12-97-S3.pdf]

**Suppl. Fig. 3**

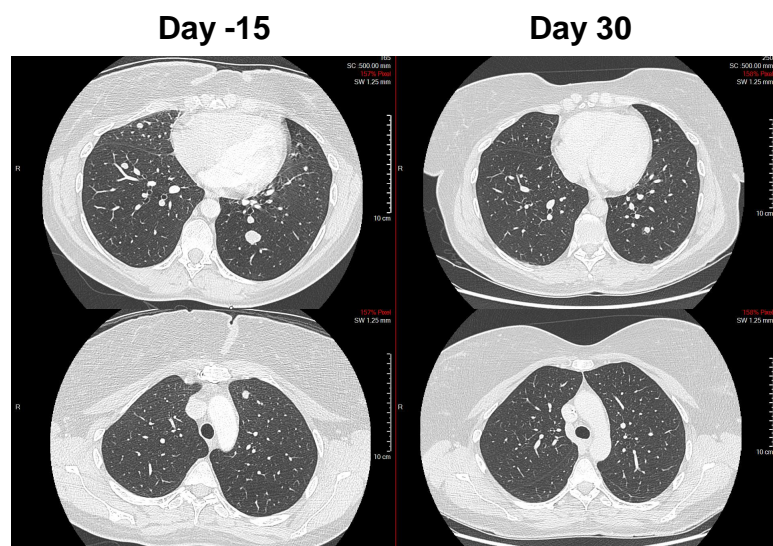

Supplement: Additional file 4: Figure S3 — Short-lived partial response in a patient from the IMP321 group. Panel A, baseline thorax-abdomen CT scan. Panel B, thorax-abdomen CT scan 30 days after completion of study treatment. [file 1479-5876-12-97-S4.pdf]
